# Sex differences in endothelial cell biology: cellular phenotypes and molecular regulators

**DOI:** 10.3389/fphys.2026.1830278

**Published:** 2026-05-08

**Authors:** Alexandra Pislaru, Tara L. Haas

**Affiliations:** 1 Department of Biology, York University, Toronto, ON, Canada; 2 School of Kinesiology and Health Science, York University, Toronto, ON, Canada

**Keywords:** endothelium, epigenetics, sex dimorphism, transcriptome, vascular disease

## Abstract

Endothelial cells (EC) line all blood vessels and are central to maintaining vascular homeostasis by regulating barrier integrity, angiogenesis, inflammation and other key processes. Although EC share core functions, they exhibit substantial heterogeneity across vascular beds and tissues, reflecting differences in local metabolic and microenvironmental conditions. Emerging evidence indicates that endothelial biology is also shaped by sex. Male and female EC differ in multiple aspects of cellular physiology, including metabolic activity, nitric oxide signaling, angiogenic capacity, oxidative stress responses, inflammatory signaling, and susceptibility to senescence. In this review, we summarize the current knowledge of these sex-dependent EC phenotypes and discuss how they vary by vascular location, as well as across age and disease context. Increasing evidence suggests that these differences arise from a complex interplay between sex hormones, sex chromosome complement, epigenetic regulation, and transcription factor networks. We discuss recent advances in understanding these mechanisms and their contributions to EC heterogeneity. Recognizing sex-specific EC functional characteristics and vulnerabilities is critical for a comprehensive understanding of vascular physiology and pathology, with important implications for improved knowledge of vascular disease mechanisms and development of more precise, sex-informed therapeutic strategies.

## Introduction

Endothelial cells (EC) form a continuous monolayer lining all blood vessels and they share core functions essential for vascular homeostasis, including maintenance of barrier integrity, vascular tone, and suppression of inflammatory and thrombotic signaling (Pluta et al., 2025). Despite their similar features, EC display considerable heterogeneity in their phenotype and function across different regions of the vascular tree. Macrovascular EC from arteries and veins are subject to vastly different hemodynamic factors that in turn influence cellular structure, junction formation and functions (Gimbrone et al., 1999; Kosyakovsky et al., 2025). Microvascular EC are strongly influenced by the local tissue microenvironment and adapt to meet tissue-specific metabolic demands (Aird, 2007). Transcriptome-level characterization of EC sub-populations has also revealed substantial tissue-specific heterogeneity in capillary EC signatures (Kalucka et al., 2020; Niu et al., 2024), indicating that local tissue environments drive distinct EC phenotypes. In addition to these location-specific phenotypes, substantive sex-specific differences in EC signaling, stress responses, and adaptive capacity have been reported (Martin et al., 2008; Green et al., 2016; Stanhewicz et al., 2018; Dearing et al., 2022), providing strong evidence that EC in males and females do not respond identically to physiological or pathological challenges.

The prevalence, severity and clinical outcomes of numerous vascular diseases display sex disparities (Szadvári et al., 2023; Spiering et al., 2025). Males display vulnerability to cardiometabolic complications from obesity (Brettle et al., 2022; Kim et al., 2025). Pre-menopausal women often exhibit lower incidence of atherosclerotic disease, including coronary artery disease and myocardial infarction; however, this apparent protection is diminished or even reversed post-menopause, with elevated risk of atherosclerosis and both pulmonary and systemic hypertension (AlSiraj et al., 2019; Man et al., 2020; Sakkers et al., 2023; Fasero and Coronado, 2025), and some neurovascular-associated diseases including Alzheimer’s disease (Briceno Silva et al., 2024). It is plausible that sex-specific regulatory mechanisms that modulate EC phenotype can contribute to overall susceptibility to these vascular pathologies. However, the underlying mechanisms for these diseases are multi-cellular in origin, thus the extent to which EC phenotype specifically drives disease risk is not well-established.

A comprehensive understanding of sex-dependent EC biology is therefore essential for identifying mechanisms underlying vascular pathophysiology and for advancing more precise, sex-informed approaches to cardiovascular research and therapy. This review will provide an overview of reported functional sex differences in EC and discuss current understanding of the molecular mechanisms underlying these distinct behaviors.

## Sex-biased functional differences in EC

Hallmarks of healthy endothelial cells include the appropriate balance of vasodilator and vasoconstrictor factors, flow (shear stress) sensitivity, formation of a monolayer that limits leakage of plasma constituents, the presentation of a non-inflammatory cellular interface with the blood, and the capacity to proliferate and form new capillaries upon stimulation. We will review current evidence for sex differences in these fundamental physiological attributes of EC. Key findings discussed below also are summarized in **Table 1**.

**Table 1:** Endothelial sex differences in cellular functions and transcriptome signatures.

Species	Cell Type	Experimental Approach	Main Sex Differences	References
Nitric Oxide Production and Vasodilation				
Human	HUVEC	Functional / molecular assays	F > eNOS expression and activity;	Cattaneo et al., 2017, 2021
Mouse	Carotid arteries	Functional assay	M > serotonin-induced constriction	Lamping & Faraci, 2001
Rat	Skeletal muscle microvessel EC	Transcriptome	F > PDE3B (phosphodiesterase isoform) M > PDE1A (phosphodiesterase isoform)	Wang et al., 2010
Human	HUVEC	Transcriptome/ in vitro assays	F > shear-stress gene expression alterations	Lorenz et al., 2015
Proliferation and Angiogenesis				
Mouse	EC from 5 organs (aged mice)	Transcriptome (RNA-seq)	F > proliferation M > inflammation and immune signature	Huang et al., 2021
Mouse	Aortic EC (OCT4 deficiency)	Functional	M > wound healing, neovascularization, and ischemic recovery	Shin et al., 2025
Human	Endothelial progenitor cells	Functional	F > Circulating endothelial progenitor cells, contributing to repair and vascularization	Fadini et al., 2008
Human	Pulmonary microvascular EC	Transcriptome / proteome	F > Thrombospondin-1; Secretome enhances EC sprouting M > Proliferation	Kostyunina et al., 2024; Hayward-Piatkovskyi et al., 2023
Human	HUVEC	Transcriptome/ in vitro assays	F > Proliferation, Migration; tube formation and viability; enhanced angiogenic potential M > Autophagy	Addis et al., 2014; Baggio et al., 2022; Lorenz et al., 2015; Boscaro et al., 2020
Human	HUVEC	Viability and angiogenesis assays	F > Viability, angiogenic capacity M > Oxidative stress	Zhang & Lingappan, 2017
Human	HUVEC	Functional / molecular assays	F > Migration-dependent angiogenesis M > eNOS-independent proliferation	Cattaneo et al., 2017, 2021
Human	Muscle and serum	Molecular assays	F > VEGF levels	Lindholm et al., 2014; Malamitsi-Puchner et al., 2000
Metabolism and Mitochondrial Function				
Mouse	Adipose EC	Transcriptomics	F > oxidative phosphorylation capacity	Rudnicki et al., 2023
Mouse	Mesenteric artery EC	Mitochondrial assays	F > Mitochondrial mass, fusion, and membrane potential F > enhanced mitochondrial function (estrogen-dependent)	Damacena de Angelis et al., 2022
Rat	Cerebral arteries	Mitochondrial respiration assays	F > mitochondrial oxidative respiration	Rutkai et al., 2015
Mouse / Human	Pulmonary and aortic EC	Mitochondrial function assays	M > mitochondrial potential in aortic EC M > mitochondrial function and higher proliferative capacity in pulmonary EC	Zemskova et al., 2020; Shin et al., 2024
Human	Adipose/skeletal EC	Transcriptomics	F > genes involved in fat oxidation (eg. CD36)	Niu et al., 2024
Oxidative Stress, Inflammation, Senescence				
Human	HUVEC	ROS assays	M > basal free ROS	Miller et al., 2007
Mouse/ Human	Aortic EC	ROS assay/ Transcriptomics	F > cellular ROS levels and apoptosis, inflammation genes in atherosclerosis M > Endothelial to mesenchymal rates	Shin et al., 2024
Mouse / Human	EC (+ H2O2)	Apoptosis assays	M > apoptosis	Norton et al., 2019
Rat/Pig/Human	Carotid artery EC, Coronary arteries and VSMCs	Molecular / antioxidant enzyme assays	F > antioxidant enzyme mRNA, e.g., SOD1 M > NADPH oxidase subunits; more oxidative	Morales et al., 2015; Kander et al., 2017; Wong et al., 2015
Human	HUVEC (+ox-LDL)	In vitro assays	F > Mitochondrial fusion, autophagy, higher resilience M > mitochondrial fission, ROS production, apoptosis	Cittadini et al., 2026
Human	HUVEC	Inflammatory stimulation	F > SOD2 mRNA in response to stress M > more inflammatory activation	Doro et al., 2024; Najjar et al., 2023
Human	HAEC	In vitro assays	M > activation of NF-κB	Najjar et al., 2023
Human	HUVEC	In vitro assays	F > senescent response to stress M > earlier senescence	Quarder et al., 2026
Human/Rat	Brain, aorta, skeletal EC	Transcriptome	M > adhesion molecule expression (PECAM1, ICAM1, VCAM1)	Hunter et al., 2019; Huxley et al., 2018
Human	Aortic EC/ HUVECs	Inflammatory stimulation/ In vitro assays	M > basal phosphorylation of NFκB p65 subunit F > NFκB activation in response to inflammatory stimulation	Najjar et al., 2023
Cardiometabolic Diseases				
Human	Atherosclerotic plaques	In situ / transcriptional profiling	F > TGFβ-driven EndMT, ECM remodeling M > Inflammatory signaling	Sakkers et al., 2026
Human	Carotid plaque EC	Single-cell RNA-seq	F > EndMT markers, oxidative stress response, autophagy M > angiogenesis, proliferation, migration	Sukhavasi et al., 2025
Human	Coronary artery (CAD patients)	Function/ Transcriptomics	F > sensitivity of artery to Ach M > pro-senescent, pro-inflammation profile	Mury et al., 2024
Mouse	Adipose EC (high fat diet)	Transcriptomics Histology	F > proliferation and chromatin remodeling; higher capillary density and EC content F > VEGF-A levels M > inflammatory and senescence-associated pathways	Rudnicki et al., 2023; Rudnicki et al., 2018
Mouse	Hippocampal neurovascular EC (high fat diet)	Single-cell transcriptomics	F > cAMP signaling pathways; healthier transcriptomic profiles M > insulin signaling and resistance; focal adhesion, PI3K-Akt, and Ras	Norman et al., 2025

F, female; M, male; >, has greater.

### Nitric oxide and other endothelial-derived vasodilatory factors

Nitric oxide (NO), produced by endothelial nitric oxide synthase (eNOS) is a major regulator of EC phenotype. NO contributes to shear stress-signaling, moderation of vascular tone, capillary permeability and angiogenesis while also serving to limit thrombosis and inflammation (Garcia and Sessa, 2019; Lundberg and Weitzberg, 2022). Thus, NO is often considered as a central marker of healthy EC, with impairment in NO production (or bioavailability) being a hallmark of dysfunction and increased susceptibility to vascular disease (Cyr et al., 2020).

Sex bias in NO production and signaling is supported by evidence that female HUVEC exhibit higher basal eNOS activity and increased NO production compared with male cells (Cattaneo et al., 2021). Consistent with these cellular findings, some vascular studies show that NO production contributes to enhanced vasodilator and/or lowered vasoconstrictor responses in females. For example, serotonin-induced constriction of carotid arteries was attenuated in female compared to male mice, with genetic deletion of eNOS eliminating this advantage (Lamping and Faraci, 2001). Estradiol influences multiple aspects of the eNOS pathway, including promoting greater mRNA, as well as Akt-dependent eNOS activation [as reviewed by (Stanhewicz et al., 2018)]. Sex differences in NO signaling may also arise from variation in downstream cyclic nucleotide pathways. In microvascular EC, intrinsic differences in phosphodiesterase expression and activity alter the breakdown of cyclic guanosine monophosphate (cGMP), a major mediator of NO signaling, thereby influencing the magnitude and duration of NO-dependent responses in male and female EC (Wang et al., 2010).

Enhanced responsiveness of females to endothelial-dependent vasodilators [as reviewed by (Stanhewicz et al., 2018)] extends beyond the NO pathway and also reflect enhanced levels of other EC-derived relaxation factors (i.e. endothelial-derived hyperpolarizing factor, prostaglandins, epoxyeicosatrienoic acids) or the lowered production of (or response to) vasoconstrictor factors such as endothelin-1 and angiotensin 2. Although sex differences in these factors have been investigated less extensively as compared to the NO pathway, data from both rodent and human studies support the existence of sex-biased signaling [as reviewed by (Pabbidi et al., 2018)]. Together, these studies provide substantive evidence that vasodilator: vasoconstrictor balance is maintained to a greater extent in females compared to males, which impacts agonist-dependent as well as shear stress- and blood pressure-dependent vascular responses. Indeed, shear stress-dependent signaling appears to be greater in females than males, both *in vivo* and in cultured EC (Levenson et al., 2001; Lorenz et al., 2015). While this may in part be due to greater capacity to produce vasodilators, female EC appear to evoke a broad pattern of shear-stress gene expression alterations that include downregulation of endothelin-1 (Lorenz et al., 2015). Overall, these sex differences are postulated to influence susceptibility to vascular disease such as hypertension and thus are relevant when considering optimal therapeutic approaches (Pabbidi et al., 2018).

### Angiogenesis

EC mostly remain quiescent in the adult but during times of changing metabolic demands, tissue growth or repair, capillary EC undergo angiogenesis to expand or re-build the capillary network (Carmeliet and Jain, 2011; Haas and Nwadozi, 2015; Corvera et al., 2022). During sprouting angiogenesis, capillary EC transition to an active phenotype characterized by proliferation and migration in response to activating signals that typically originate from the parenchymal cells (Abranches et al., 2015; Potente and Carmeliet, 2017). This functional shift is supported by metabolic reprogramming that involves substantial upregulation of glycolytic activity (Eelen et al., 2018). Concurrently, mitochondrial metabolism and fatty acid oxidation support redox balance and nucleotide synthesis, which are required to sustain EC proliferation (Schoors et al., 2015; Eelen et al., 2018; Kalucka et al., 2018). These metabolic adaptations sustain the sprouting process, with cells reverting to a quiescent phenotype as the new vessels mature and stabilize.

Sex differences in endothelial angiogenic capacity are evident *in vivo* across several physiological and metabolic contexts. In adipose tissue, female mice exhibit greater capillary density, EC proliferation and endothelial marker expression under normal-chow and high-fat diet conditions, which is associated with improved vascular remodeling and a healthier adipose phenotype (Rudnicki et al., 2018, 2023). Consistent with enhanced vascular growth potential, females also display higher levels of circulating endothelial progenitor cells, which contribute to vascular repair and neovascularization (Fadini et al., 2008). Higher VEGFA levels have been reported in females in muscle, serum and in adipose tissue of female mice (Malamitsi-Puchner et al., 2000; Lindholm et al., 2014; Rudnicki et al., 2018). However, the extent to which parenchymal tissue VEGFA contributes to the proliferative and angiogenic advantage observed in female EC remains unclear, particularly since this proliferative phenotype can be retained in female cultured cells in the absence of external pro-angiogenic stimuli (Rudnicki et al., 2023). Additionally, male and female EC secrete distinct profiles of angiogenic factors that can influence endothelial behavior in a sex-specific manner (Hayward-Piatkovskyi et al., 2023). Interestingly, VEGF signaling pathway inhibitors are reportedly more efficient in controlling tumor growth in females (Cignarella et al., 2022). While the underlying mechanisms require investigation, it is tempting to speculate that sex differences in angiogenesis signaling may underpin these outcomes.

Functional studies using cultured cells support the existence of intrinsic sex differences in angiogenic capacity. Although it has been suggested that female EC rely more heavily on migration-dependent mechanisms, whereas male cells depend more on proliferative expansion during angiogenic growth (Cattaneo et al., 2017), the more consistent observation in cultured cells is that of higher proliferation in female EC. For example, female murine adipose EC retain a higher proliferative phenotype than male EC cultured cells, in the absence of external pro-angiogenic stimuli (Rudnicki et al., 2023). Female human EC also commonly display greater viability, migration, and angiogenic potential compared with male cells (Addis et al., 2014; Lorenz et al., 2015; Boscaro et al., 2020; Baggio et al., 2022).

Together, these studies suggest that angiogenic outcomes are influenced by sex, but the magnitude of these differences vary dependent on the tissue and the pathophysiological context.

### Energy metabolism and mitochondrial function

Metabolic pathways fuel basic cellular processes and regulate angiogenesis. EC rely predominantly on glycolysis for ATP production, while mitochondrial-linked pathways such as fatty acid oxidation and the tricarboxylic acid cycle support redox homeostasis, and nucleotide production (De Bock et al., 2013; Schoors et al., 2015; Eelen et al., 2018). Together, these various metabolic programs support the basal functions of quiescent cells as well as the energy-intensive angiogenesis process, an important feature of vascular adaptation (Potente and Carmeliet, 2017). Investigations of EC suggests that mitochondrial function differs between sexes, although the reported direction and magnitude of these differences vary across studies.

Greater oxidative metabolic capacity in female EC has been documented in rat, human, and mouse cells derived from multiple vascular beds, with studies reporting increased mitochondrial respiration, oxidative phosphorylation capacity, mitochondrial mass, and membrane potential that collectively support enhanced oxidative metabolism (Rutkai et al., 2015; Damacena de Angelis et al., 2022; Rudnicki et al., 2023). Structural differences in mitochondrial organization, such as increased mitochondrial fusion, may further promote efficient oxidative phosphorylation and metabolic adaptability in female EC (Khalifa et al., 2017; Damacena de Angelis et al., 2022; Rudnicki et al., 2023). Enhanced mitochondrial Ca2+ handling in female EC also was shown to influence endothelial NOS activity and vasodilatory signaling (Damacena de Angelis et al., 2022). Interestingly, CD36, a fatty acid transporter involved in lipid uptake, is expressed at higher levels in female compared to male human EC across multiple tissues including skeletal muscle and white adipose depots (Niu et al., 2024), which could support greater fatty acid uptake to maintain higher rates of lipid oxidation. The enhanced mitochondrial function and oxidative metabolic activity generally observed in female EC may support enhanced oxidative stress maintenance and nucleotide synthesis that are required for proliferation. These advantages thus may help preserve endothelial function and promote angiogenic potential.

Although the preponderance of data support the conclusion that female EC have higher oxidative metabolic activity, there are several studies that report the opposite, with male EC (both human and mouse) having higher mitochondrial membrane potential, basal mitochondrial respiration rate and elevated maximal respiration (Zemskova et al., 2020; Shin et al., 2024). The reasons for these disparate experimental outcomes are unclear, and cannot be easily assigned to diversity in EC origin or species, suggesting they may be associated with technical variations in experimental design such as cell culturing and experimental conditions or with assessment methodology. These inconsistencies may be diminished with the addition of more data in the future, as more studies now report sex-disaggregated data.

## Sex-divergent susceptibility to EC damage and inflammation

### Handling of oxidative stress

Cellular homeostasis relies on balancing pro-oxidant and antioxidant systems (redox) (Penna and Pagliaro, 2025). Reactive oxygen species (ROS) are generated continuously as byproducts of cellular metabolism and also serve signaling functions under physiological conditions (de Almeida et al., 2022). Oxidative stress, which develops when ROS production exceeds antioxidant defenses, causes wide-ranging cellular disruption including mitochondrial dysfunction, protein and DNA damage. Ultimately, prolonged oxidative stress can evoke senescence, an irreversible state of cell-cycle arrest (de Almeida et al., 2022) and contribute to vascular aging and the progression of cardiovascular and metabolic diseases (Jia et al., 2019; Stojanovic et al., 2025; Xu et al., 2025). Cells rely on multiple antioxidant defense systems, including enzymatic scavengers such as superoxide dismutase, catalase, and glutathione-dependent pathways, to maintain redox balance and prevent oxidative damage (Bachschmid et al., 2013; Aldosari et al., 2018; Eelen et al., 2018). Accumulating evidence across multiple cell types suggests that females exhibit greater resilience to oxidant-induced stress than males, likely due to enhanced antioxidant defenses and more efficient mitochondrial efficiency (Borrás et al., 2003; Vina et al., 2011; Capllonch-Amer et al., 2014; Ventura-Clapier et al., 2017; Martínez de Toda et al., 2023).

Sex-dependent differences in redox balance have been reported in vascular cells (Robert, 2023), however direct evidence in EC remains limited and, in some cases, context-dependent. Studies in vascular tissues and smooth muscle cells suggest that females may exhibit higher expression of antioxidant enzymes such as superoxide dismutase (Morales et al., 2015), whereas males show increased expression of NADPH oxidase subunits, a major enzymatic source of ROS (Wong et al., 2015; Kander et al., 2017). It also was reported that female EC specifically have higher glutathione (GSH) levels, suggesting enhanced oxidant buffering capacity through the GSH/GSSG pathway (Cruikshank et al., 2024). However, given the variability in experimental models and the contribution of non-endothelial cell types in many studies, these findings should be interpreted with caution.

Functional studies also provide support for improved tolerance to increased ROS production and oxidant exposure in female EC. Under hyperoxic conditions, male HUVEC exhibit greater ROS production, compared with female cells (Zhang and Lingappan, 2017). Exposure to hydrogen peroxide induces greater apoptosis in male EC than in female EC (Norton et al., 2019), further supporting enhanced resistance to oxidative damage in females. Redox regulation also varies across endothelial beds, with sex- and tissue-specific differences in protein expression of antioxidants such as superoxide dismutase 2 (SOD2). In human aortic EC, SOD2 expression is higher in male cells, whereas in HUVEC, SOD2 expression is higher in female cells, contributing to distinct redox states and profiles between sexes (Najjar et al., 2023). Consistent with these findings, HUVEC also show sex-specific differences in response to ROS-generating stimuli, with male cells exhibiting greater injury induced by oxidized LDL, mitochondrial fission, reactive oxygen species production, and apoptosis, and impaired angiogenic capacity compared to female EC, which retain greater overall resilience (Zhang and Lingappan, 2017; Norton et al., 2019; Cittadini et al., 2026).

However, the apparent female advantage in ROS handling and antioxidant capacity is not present in some pathophysiological contexts (Kander et al., 2017). In atherosclerotic models, female aortic EC exhibit increased ROS accumulation compared with male cells (Shin et al., 2024). Sex-specific susceptibility to ROS-mediated stress may contribute to endothelial dysfunction in human microvascular studies, as superoxide-mediated impairment of nitric oxide-dependent vasodilation has been shown to be more pronounced in female chronic e-cigarette users (Halstead et al., 2023). These findings suggest that sex-specific endothelial responses to ROS may be highly dependent on the source of ROS and microenvironmental influences.

### Inflammation and senescence

Inflammatory signaling pathways in EC are typically activated in response to cellular stress and may persist during chronic disease states. In addition to cytokine stimulation, pro-inflammatory signaling can be initiated downstream of EC mitochondrial dysfunction and increased ROS generation, particularly through the transcription factor NFκB (Csiszar et al., 2008; Forrester et al., 2020). Nuclear-localized NFκB promotes the expression of endothelial adhesion molecules such as vascular cell adhesion molecule-1 (VCAM-1), intercellular adhesion molecule-1 (ICAM-1), and E-selectin, facilitating leukocyte recruitment, adhesion, and transmigration across the vascular wall (Collins et al., 1995). Through these mechanisms, oxidative stress and mitochondrial dysfunction can shift EC toward a pro-inflammatory phenotype, promoting senescence and disrupting vascular homeostasis (Peng et al., 2019; Xiao et al., 2021; Grossini et al., 2025; Penna and Pagliaro, 2025). Consequently, EC inflammation represents a key transition point between normal physiological regulation and the progression toward vascular disease.

Several studies have demonstrated sex-dependent regulation of NFκB signaling, although there are substantial influences of EC type and inflammatory context. For example, male-derived human aortic EC displayed higher basal phosphorylation of the NFκB p65 subunit compared with female-derived cells whereas no basal sex differences were observed in HUVEC or HMVEC (Najjar et al., 2023). In response to stimulation with the pro-inflammatory cytokine TNFα, female HUVEC displayed greater increases NFκB activation compared with male counterparts, whereas the opposite pattern was observed in HMVEC (Najjar et al., 2023).

Inflammatory responses that arise secondary to cellular stress or tissue damage often appear less pronounced in females, consistent with their greater resistance to oxidative stress (Martínez de Toda et al., 2023). For example, TNFα stimulation elicits exhibit a broader increase in adhesion molecule expression (PECAM1, ICAM1, E-selectin) in male compared with female human brain EC (Hunter et al., 2019). Consistent with these findings, adhesion molecules ICAM1 and VCAM1 were higher in rat male ECs from the aorta and skeletal muscle ECs compared to those from females (Huxley et al., 2018), suggesting enhanced leukocyte-endothelial interactions in males. Likewise, male EC (human aorta) demonstrate stronger activation of pro-inflammatory NFκB signaling compared to female cells (Najjar et al., 2023). Cultured adipose EC from mice also show elevated inflammatory responses in males both at baseline and following TNFα stimulation compared with females (Rudnicki et al., 2023). Consistent with this, human studies demonstrate that females exhibit accelerated resolution of inflammation, characterized by reduced leukocyte activation, associated with protection from inflammation-induced EC dysfunction compared to males (Rathod et al., 2016). However, this suppressed inflammatory response of female EC may be stimulus- or context-specific. For example, female HUVEC showed a greater cytokine production than male cells when challenged with lipopolysaccharide (Doro et al., 2024). Together, these findings suggest that sex differences in inflammatory signaling are prevalent and likely contribute to the distinct trajectories of male and female EC during aging and development of cardiometabolic disease.

Although senescence, or irreversible exit from the cell cycle, is a fundamental aspect of aging, premature cellular senescence is triggered by mitochondrial dysfunction, chronic oxidative stress and inflammation (Stojanovic et al., 2025) and contributes to EC dysfunction through irreversible growth arrest and the acquisition of a pro-inflammatory secretory phenotype (Mury et al., 2024). DNA repair-deficient mouse models, which detect age-related accumulation of senescent cells, highlight a greater burden of senescent cells in males compared to females across most of the lifespan (Yousefzadeh et al., 2020). Consistent with this, EC in the coronary arteries of female patients with coronary artery disease display substantially lower signatures of senescence-associated cellular pathways (Mury et al., 2024). Cultured female EC also exhibit reduced markers of replicative senescence compared with male EC (Quarder et al., 2026). However, these differences are not uniform across conditions, as female EC may exhibit increased susceptibility to certain stress-induced senescence responses, including enhanced inflammatory or stress-associated signaling following irradiation injury (Quarder et al., 2026). These findings suggest that sex differences in EC senescence tend to globally protect females, yet this advantage is not maintained in all disease contexts.

### Linkage to vascular disease

The sex-biases in EC in functions (vasodilation, metabolism, angiogenesis) and responses to stressors (redox balancing, inflammatory signaling, senescence) described in the above sections contribute broadly to establishing divergent vascular outcomes to the same stimuli and to shaping susceptibility to vascular dysfunction and disease (Stanhewicz et al., 2018; Boscaro et al., 2020; Cookson and Sherlock, 2023; Robert, 2023; Quarder et al., 2026). There are several examples of cardiovascular-metabolic diseases where EC sex differences have been linked to disease outcomes.

In mouse models, obesity has been shown to severely inhibit adipose tissue angiogenesis in males, which exacerbates adipose dysfunction and systemic metabolic disturbances (Molnar et al., 2005; Aoqui et al., 2014; Corvera and Gealekman, 2014; Hasan et al., 2025). In contrast, obese female mice retain superior angiogenic capacity in the white adipose tissue, which is associated with improved vascular remodeling and a healthier adipose phenotype (Rudnicki et al., 2018, 2023). Adipose EC from males exhibit heightened inflammatory gene expression and signaling compared to females, particularly under obesogenic conditions and during aging (Huang et al., 2021; Rudnicki et al., 2023). Obesity was shown to amplify the pro-inflammatory pathways (including STAT3, interferon, and RAGE signaling) in adipose EC from male mice, indicative of increased EC dysfunction (Hasan et al., 2025). Interestingly, female sex hormones do not appear to drive the sex-specific mechanisms of obesity-related endothelial dysfunction, as ovariectomy in female mice did not provoke EC impairment (Barris et al., 2025). Consistent with this, human studies demonstrate that females exhibit accelerated resolution of inflammation, characterized by reduced leukocyte activation, associated with protection from inflammation-induced EC dysfunction compared to males (Rathod et al., 2016).

Sex-distinct immune responses in atherosclerosis have been reported, with males exhibiting greater inflammatory signaling, and females mounting stronger adaptive immune responses (Fairweather, 2015). However, in some atherosclerotic models, female aortic EC exhibit increased apoptosis compared with male cells (Kander et al., 2017). In human atherosclerotic plaques, female EC exhibit enhanced transforming growth factor-β (TGF-β)-driven endothelial-to-mesenchymal transition (EndMT) and extracellular matrix remodeling, whereas male EC show stronger inflammatory signaling (Sakkers et al., 2026). Recent single-cell transcriptomic analysis of EC from carotid artery atherosclerotic plaque supported these findings, by identifying distinct female EC subpopulations that displayed enrichment in genes associated with oxidative stress responses, autophagy, and EndMT markers (Sukhavasi et al., 2025).

The sex-biased but variable directionality of protection associated with certain types of disease suggests that the type of stressor, or vascular bed-specific factors, may modify sex-dependent redox and inflammatory responses. For example, it has been postulated that atherosclerosis-associated chronic vascular inflammation generates high levels of ROS through NADPH oxidases (Poznyak et al., 2020), which male cells may be better equipped to dissipate. In contrast, during obesity, endothelial stress is largely driven by lipid and glucose overload, and the greater mitochondrial efficiency of female EC may facilitate better adaptation to the associated increased oxidant stress (Symons and Abel, 2013; Burgos-Morón et al., 2019). However, much remains to be established with respect to defining the factors that drive these EC sex differences. Expanding this knowledge will be an important step in understanding disease susceptibility and developing appropriate therapeutic strategies.

## Sex-dependent EC transcriptome programing as a driver of cellular phenotype

**Figure 1:**
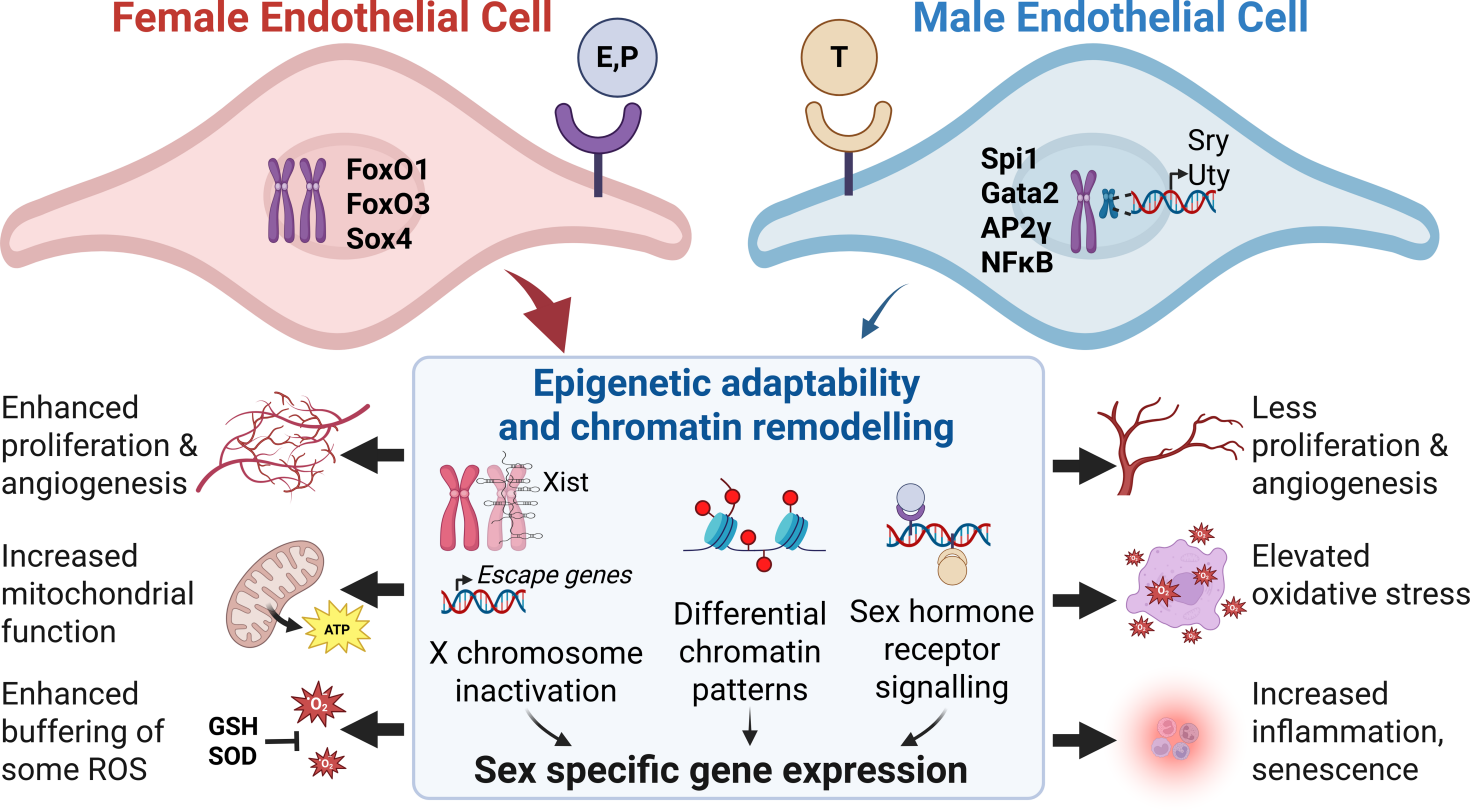
Sex-specific regulation of endothelial cell phenotype. Male and female EC exhibit distinct transcriptional programs driven by sex chromosome genes, hormone signaling, and epigenetic regulation. Female EC cells display a generally healthier phenotype, characterized by enhanced angiogenesis, improved mitochondrial function, and greater resistance to oxidative stress, likely supported by greater chromatin adaptability and enrichment of transcriptional regulators such as FoxO1, FoxO3, and Sox4. In contrast, male EC show increased activity of inflammatory transcription factors (Spil, Gata2, AP2γ, NFκB) and Y- chromosome genes (Sry, Uty), contributing to elevated oxidative stress, inflammation, and reduced proliferative capacity. E, estradiol; P, progesterone; T, testosterone; GSH, glutathione; SOD superoxide dismutase. See text for further details. Schematic created in BioRender. https://BioRender.com/cpxw9hl.

The sex differences in EC phenotype and function that establish the balance between health and disease susceptibility arise from a complex interplay of genetic, hormonal and epigenetic influences that is challenging to unravel (see **Figure 1**) (Szadvári et al., 2023; Spiering et al., 2025). Foremost, functional differences in male and female EC reflect broadly different programming of gene transcription.

The expanding availability of transcriptomic investigations that include samples from both sexes is providing unprecedented opportunities for unbiased assessment of EC sex-distinct transcriptomic signatures across multiple tissues. Recently, a comprehensive single-cell transcriptomic atlas of over 210,000 human EC across 38 regions from 24 tissues revealed extensive heterogeneity in gene expression, phenotype, metabolism, and transcriptional regulation among organ-specific EC populations (Niu et al., 2024). The dataset demonstrated tissue-independent and tissue-specific sex differences, with significant sex-biased gene expression detected in organs including the aorta, skin, visceral adipose tissue, brain, liver, and skeletal muscle. Across tissues, female endothelial signatures were enriched for pathways related to adhesion, migration, vascular development, and TGF-β signaling, whereas male signatures were more strongly associated with immune signaling, antigen processing, and stress-response pathways (Niu et al., 2024).

Similarly, interrogation of the Tabula Muris dataset, which includes single cell transcriptomic data from multiple mouse tissues (Tabula Muris Consortium, 2018), led to the observation that tissues such as the aorta, brain, and lung contain distinct sex-specific endothelial subpopulations, whereas organs including adipose tissue, heart, and kidney exhibit more nuanced, mixed distributions of male and female EC (Paik et al., 2020). In the brain and lung, female EC were enriched for pathways related to ribosome dynamics and oxidative phosphorylation. The study identified Lars2, a non-sex-linked gene encoding mitochondrial leucyl-tRNA synthetase, as consistently and highly expressed in male EC but low and variable in female EC at both the mRNA and protein levels, with expression largely endothelial-specific and enriched in male tissues (Paik et al., 2020), although the functional significance of this differential expression is not yet known.

Other studies have utilized bulk RNA-sequencing to analyze sex differences of EC originating from a single vessel or tissue. Integrated transcriptomic and proteomic analyses of human pulmonary microvascular EC, under both normoxic and hypoxic conditions, identified sex-specific regulatory signatures, with female cells exhibiting higher expression of the anti-proliferative matrix protein thrombospondin-1 and altered pathways controlling cell cycle progression (Kostyunina et al., 2024). Sex-based comparisons of the transcriptomes of EC from the hippocampal neurovascular unit of obese mice identified males having higher insulin signaling and resistance, focal adhesion, PI3K-Akt signaling and Ras signaling, while females EC were enriched in cAMP signaling pathway (Norman et al., 2025). Several transcriptomic analyses (both human and mouse EC) have revealed a bias for female EC to be enriched in pathways related to chromatin organization and gene regulation compared to males (Hartman et al., 2020; Huang et al., 2021; Rudnicki et al., 2023).

Understanding the molecular mechanisms that regulate these sex-specific transcriptional programs is therefore critical to explaining how endothelial phenotypes diverge between males and females. Sex-divergent EC transcriptomes may be supported by differential actions of transcription factors, as there is some evidence for sex-biased expression and DNA binding patterns of transcription factors. The pluripotency factor Oct4 has emerged as an important mediator of sex-specific gene expression, partly through its regulation of Xist transcription and X-chromosome activity (Donohoe et al., 2009). Consistent with this role, deletion of Oct4 in EC produces pronounced sex-dependent transcriptional changes, affecting hundreds of X-linked genes, including many known escape genes (Shin et al., 2025). Functionally, Oct4 loss leads to divergent transcriptional responses between sexes, with male EC showing increased expression of genes associated with cell cycle progression and angiogenesis, while female EC display enhanced expression of pro-inflammatory transcripts (Shin et al., 2025). The transcription factor Forkhead BoxO1 also displays sex-biased expression patterns and is a predicted upstream regulator of the chromatin organization genes that are differentially enriched in female adipose EC (Rudnicki et al., 2023). Notably, functional studies demonstrate that FoxO1 inhibition or knockdown disproportionately affects female EC, and shifts the transcriptome signature of female adipose EC toward that of male EC (Pislaru, 2025). Together, these findings highlight that sex-specific transcriptional programs are a fundamental determinant of endothelial heterogeneity, shaping distinct phenotypic states that may underlie differential vascular function and disease susceptibility between males and females.

## Molecular bases underlying sex differences in EC phenotype

A key question is the extent to which these sex differences arise intrinsically versus via exposure to sex-hormones or other environmental stimuli. Traditionally, much research has focused on the role of sex hormones in driving these differences, and there are multiple excellent reviews on this topic (Sader and Celermajer, 2002; Stanhewicz et al., 2018; Robert, 2023). Additionally, growing evidence over the past decade has underscored the critical role of sex chromosomes and epigenetic regulation in shaping cell phenotype independent of circulating hormones. Accordingly, we will briefly discuss sex hormone-dependent effects but place greater emphasis on mechanisms beyond sex hormones that contribute to EC sex differences.

## Effects of sex hormones on EC phenotype

Sex hormones can broadly influence EC gene expression and cellular phenotype (Robert, 2023). The primary sex hormones, estrogens, progesterone, and androgens, exert their effects through binding to nuclear and membrane-associated receptors (Orshal and Khalil, 2004; Xega and Liu, 2024). Activation of these receptors can regulate cellular metabolism, proliferation, and inflammatory responses. 17β-estradiol has been demonstrated to promote multiple positive attributes of EC health by enhancing nitric oxide production and lowering of inflammation (Robert, 2023; Fontaine et al., 2025). Mechanistically, activation of estrogen receptors (ESR) or the G protein-coupled estrogen receptor (GPR30) can stimulate Akt-dependent signaling pathways that activate eNOS, leading to increased nitric oxide production and improved endothelial function (Hisamoto et al., 2001; Miller and Duckles, 2008). 17β-estradiol also promotes mitochondrial biogenesis, enhances antioxidant defenses, and improves electron transport chain efficiency in multiple cell types including EC, supporting enhanced oxidative metabolism and mitochondrial antioxidant capacity (Borrás et al., 2003; Razmara et al., 2008; Huang et al., 2025; Damacena de Angelis et al., 2022). 17β-estradiol-dependent signaling improves EC redox balance through lowering NADPH oxidase activity and upregulating antioxidant enzymes, thereby (Miller et al., 2007). Progesterone appears to exert distinct and context-dependent actions, with evidence that it can inhibit EC proliferation and promote apoptosis through progesterone receptor-mediated signaling and G1 cell cycle arrest, impair re-endothelialization *in vivo*, and also regulate EC migration (Vázquez et al., 1999; Zheng et al., 2012). Androgen receptor regulates gene expression and vascular signaling through multiple mechanisms, including modulation of NFκB-dependent inflammatory pathways, induction of adhesion molecules to promote leukocyte recruitment, and activation of eNOS phosphorylation and nitric oxide production (Torres-Estay et al., 2015). Together, these findings highlight that sex hormones influence multiple aspects of endothelial biology, including nitric oxide signaling, reactive oxygen species regulation, metabolic function and inflammatory signaling.

Sex hormone signaling reinforces differential gene expression profiles in male and female EC directly via hormone receptor-mediated transcription and indirectly through modulating the activity of other transcription factors. Comparative analysis of the sex-biased transcriptomes of human EC from neonates compared to adults was utilized in an effort to distinguish between intrinsic (neonate) versus acquired (adult) sex-biased gene expression (Hartman et al., 2020). Notably, the proportion of transcription factors among acquired sex-biased genes was higher than in the intrinsic gene set, suggesting that transcription factor-mediated regulation becomes increasingly important for shaping sex differences in endothelial gene expression over time. Pathway analysis identified estrogen as a predicted upstream regulator of sex-biased genes in adult but not neonatal female EC, nor in male EC, supporting a substantive role for 17β-estradiol in shaping the transcriptional regulation of genes in adult female EC (Hartman et al., 2020). Mechanistically, 17β-estradiol estrogen signaling can directly regulate EC transcription factor activityestrogen. For example, 17β-estradiol estrogen has been shown to modulate NFκB signaling, and female EC show stronger estrogen-dependent regulation of this signaling and higher expression of its downstream inflammatory targets (Shin et al., 2024). Moreover it was demonstrated that male EC express higher levels of the transcription factor AP2γ (Tfap2c), which controls estrogen receptor expression, implying that different estrogen receptor activity may determine male and female EC phenotypes (Shin et al., 2024). Together, these studies provide support for the importance of 17β-estradiol-dependent enforcement of female EC phenotype. Evidence supporting the roles of other sex hormones in shaping EC phenotype is lacking to date.

## Sex chromosomes and epigenetic control mechanisms

Sex hormone actions do not fully explain sex differences in EC, as exemplified by the presence of cellular sex differences in neonates, in adults in the absence of circulating sex hormones and in cultured cells (Wang et al., 2010; Arnold, 2012; Nelson et al., 2019; Hartman et al., 2020). These studies point to a role for intrinsic genetic or epigenetic mechanisms in establishing basal sex-differences. Sex divergent transcriptomes can originate from differences in gene dosage between the XX and XY chromosomes, escape of certain genes from X-chromosome inactivation, and sex-biased epigenetic regulation of autosomal gene expression (Mank, 2009; Navarro‐Cobos et al., 2020; Chlamydas et al., 2022; Thej et al., 2024; Heitzmann et al., 2025).

The Y chromosome encodes a small number of male-specific genes that may influence cellular phenotypes (Dhanoa et al., 2016; Krausz and Abrardo, 2025). Genes encoded on the Y chromosome genes do contribute to vascular health, as hematopoietic mosaic loss of the Y chromosome is associated with increased cardiovascular mortality and fibrosis (Sano et al., 2022), suggesting that Y-linked genetic pathways may promote sex-specific differences in endothelial and cardiovascular biology. The SRY gene, a Y-linked transcription factor, induces adhesion molecule expression and promotes vascular inflammation in EC, suggesting a potential role in augmenting inflammation and inflammation-associated diseases (Cai et al., 2015). Although many human Y chromosome genes have homologous counterparts on the X chromosome (Owens et al., 2024), these homologs typically do not share identical amino acid sequences, and some divergent functions have been identified. For example, the Y chromosome gene Uty, which encodes a histone demethylase, has lower catalytic activity than its X chromosome counterpart Utx (Owens et al., 2024). Uty was shown to protect against pulmonary hypertension in mice, with reduced expression associated with increased inflammatory cytokine production and endothelial death (Cunningham et al., 2022).

X chromosome gene dosage also may influence EC biology. Individuals with sex chromosome aneuploidies, such as Turner syndrome (45,X) and Klinefelter syndrome (47,XXY), present natural models of the broad effects of altered X chromosome dosage. Individuals with Turner syndrome exhibit increased cardiovascular risk, including EC dysfunction, impaired flow-mediated dilation, and increased arterial stiffness (O’Gorman et al., 2012; Silberbach et al., 2018), suggesting that reduced X chromosome dosage adversely affects vascular homeostasis. Individuals with Klinefelter syndrome display elevated cardiometabolic risk and vascular abnormalities, including EC dysfunction and increased incidence of thrombotic events (Di Mambro et al., 2010; Pasquali et al., 2013; Haymana et al., 2018; Putta Nagarajan et al., 2026), potentially reflecting the effects of an additional chromosome and altered gene dosage.

In females with typical X chromosome dosage (46,XX), one X chromosome is largely silenced through X chromosome inactivation (XCI). This is a process mediated by DNA methylation, histone modifications, and chromatin remodeling that balances X-linked gene dosage with males (Li et al., 2020; Bhattacharya et al., 2024). The long non-coding RNA Xist plays a central role in X chromosome inactivation by initiating the silencing of most X-linked genes (Ng et al., 2007). However, a subset of X-linked genes escape XCI and remain expressed from both X chromosomes, resulting in increased gene dosage in female cells (Navarro‐Cobos et al., 2020; Fu et al., 2025). Notably, the genes that escape XCI may vary developmentally, differ across tissues within the same organism, and diverge between species (Berletch et al., 2011, 2015). This variability contributes to tissue-specific and species-dependent patterns of sex-dimorphic gene expression. Several escape genes are implicated in cardiovascular and immune regulation. For example, Ddx3x has been implicated in the regulation of endothelial-to-mesenchymal transition (EndMT) (Zhang et al., 2025). Because escape from XCI results in higher gene dosage in female cells, variation or mutation in Ddx3x may disproportionately affect females and alter endothelial transcriptional programs involved in vascular remodeling (Boitnott et al., 2025). Additionally, the long non-coding RNA Xist, is implicated in cardiovascular disease through its ability to regulate inflammatory signaling and vascular cell function (Almalki, 2024). Beyond its role in XCI, Xist ribonucleoprotein complexes are strongly associated with autoimmunity and linked with the increased risk of developing autoimmune disease in females (Dou et al., 2024; McHugh, 2024). Xist also was shown to enhance ELK1 expression while suppressing the anti-proliferative factor KLF2, leading to greater proliferative capacity in human pulmonary artery EC (Qin et al., 2020). Together, these findings suggest that regulation of X-chromosome inactivation and variable escape from silencing provide mechanisms by which sex chromosome dosage can shape endothelial gene expression and contribute to sex-specific vascular phenotypes.

Epigenetic modifications (i.e. methylation, acetylation, lactylation) of histones and DNA comprising the autosomal chromosomes control chromatin structure and accessibility. These modifications therefore represent a genome-wide regulatory layer that shapes transcriptional programs underlying cellular sex differences (Allis and Jenuwein, 2016). To date, methylation modifications have received the most extensive research attention. Chromatin analyses of various cell types have typically reported that there is global hypo-methylation in males compared to females (Singmann et al., 2015; Morgan et al., 2024). The existence of epigenetic sex differences within the cardiovascular system is supported by the presence of distinct DNA methylation patterns between males and females, both globally and at specific genes linked to lipid metabolism, aging, and cardiometabolic risk (Hartman et al., 2018). An investigation of male and female mouse endothelial progenitor cells revealed differential patterns of H3K9me3 occupancy at the transcription start sites of genes associated with inflammation and angiogenesis (Thej et al., 2024). Notably, the methylation pattern in female cells was minimally affected by ovariectomy, suggesting that this epigenetic regulation is not reliant on sex hormones (Thej et al., 2024). Lactylation of histones, which is tightly associated with glycolytic flux (Zhang et al., 2019), is also attractive to consider from the perspective that it may integrate sex differences in EC metabolism with chromatin organization. Overall, however, much remains to be explored regarding the existence of sex-distinct epigenetic marks in EC and the extent to which these establish sex dimorphic gene expression profiles.

## Summary and future perspectives

Understanding sex differences in EC biology has important implications for both basic research and clinical practice. The mounting evidence that male and female EC differ in metabolism, angiogenic capacity, stress responses and inflammatory signaling support the concept that sex-specific endothelial programs influence vascular repair, angiogenesis, and susceptibility to cardiovascular disease (summarized in **Figure 1**). Furthermore, these fundamental biological differences suggest that drug treatments for cardiovascular disease may manifest sex-specific efficacies. This highlights the importance of considering sex as a biological variable in preclinical studies and clinical trials, while also pointing to the opportunity to leverage sex-specific EC physiological responses to enhance vascular health.

Beyond intrinsic chromosomal and transcriptional differences, endothelial function is influenced across the lifespan by sex hormones, aging, and mosaic loss of the Y chromosome, which can alter cellular phenotypes in men. These factors are further complicated in populations utilizing sex hormone therapy, underscoring the importance of personalized approaches that consider both biological sex and sex hormone influences in predicting vascular risk and response to therapy. Future research integrating longitudinal studies, single-cell transcriptomics, and functional assays will be critical for identifying sex- and age-specific therapeutic targets, optimizing cardiovascular interventions, and ultimately improving outcomes across diverse populations.
